# Non-cotton swab sample collection may not affect salivary melatonin assay results

**DOI:** 10.1186/s40101-018-0178-6

**Published:** 2018-06-18

**Authors:** Tomoaki Kozaki, Yuki Hidaka

**Affiliations:** 1 Fukuoka Women’s University, 1-1-1 Kasumigaoka, Higashi-ku, Fukuoka, 813-8529 Japan; 2 Kyushu University, 4-9-1 Shiobaru, Minami-ku, Fukuoka 815-8540 Japan

**Keywords:** Saliva sample, Melatonin, Non-cotton swab

## Abstract

**Background:**

Salivary melatonin levels have been analyzed in many research fields, including physiological anthropology. Although various devices have been utilized for saliva collection, cotton swabs are among the most common. However, previous studies have reported that cotton swabs may interfere with melatonin assay results, whereas synthetic swabs may not. These studies compared only mean melatonin levels between passive and synthetic-polymer swab collection methods but did not evaluate relative and proportional biases. Our study examines the effects of using swabs made of materials other than cotton, such as polypropylene–polyethylene polymer, on salivary melatonin assay results using a Bland–Altman (BA) plot. The effects of the saliva collection method were analyzed using two concentrations of melatonin, lower (< 6 pg/ml) and higher (> 6 pg/ml), because the threshold of dim light melatonin onset was lower than 6 pg/ml in many studies.

**Results:**

Differences detected between passive and polypropylene–polyethylene polymer swab methods of saliva collection were not significant in both lower (< 6 pg/ml) and higher (> 6 pg/ml) melatonin levels detected. All correlations between the collection methods were significant, and 95% confidence intervals for differences in melatonin levels in all samples detected using passive and non-cotton swab saliva collection methods included zero in the BA plots. Averages and differences between non-cotton and passive saliva collection obtained from the BA plots were not significantly correlated at lower and higher melatonin levels.

**Conclusions:**

Our findings demonstrate that swabbing methods, including the use of polypropylene–polyethylene polymer, do not affect salivary melatonin assay results. Therefore, the authors suggest that polypropylene–polyethylene polymer swab methods are appropriate for the assessment of dim light melatonin onset and dose response of the circadian system to light.

## Background

Melatonin levels produced by the pineal gland [1, 2] are hypothesized to regulate circadian phases in humans, particularly the onset of melatonin secretion under dim light conditions (dim light melatonin onset or DLMO) [3, 4], and are used to assess the dose response of various stimuli on the circadian system, such as light [5–8]. The salivary melatonin assay has recently been developed as an alternative to measuring blood melatonin levels, because salivary and blood melatonin levels are correlated, e.g., the coefficient of correlation between saliva and plasma samples for DLMO was *r* = .70 and for melatonin acrophase was *r* = .55 [9]. Furthermore, the collection of salivary samples is less intrusive and more convenient for study participants than the collection of urine or blood samples. Therefore, salivary melatonin levels are obtained in many research fields, including physiological anthropology [10–14].

Although various simple devices have been used for saliva collection, cotton swabs are among the most common. However, previous studies have reported that cotton swabs may affect the results of the salivary melatonin assay [15, 16]. Kozaki et al. [16] examined the effect of cotton swab collection on the results of the salivary melatonin assay using enzyme immunoassay. In their study, saliva samples were directly collected in plastic tubes using plastic straws, and the sample was subsequently pipetted onto cotton swabs (cotton swab saliva collection) as well as into clear sterile tubes (passive saliva collection). The saliva samples were separately analyzed at lower (< 6 pg/ml) and higher (> 6 pg/ml) melatonin levels, because DLMO thresholds in many studies have been set below 6 pg/ml. Although Bland–Altman (BA) plots [17] indicate that the cotton swab method causes relative or proportional bias in assay results at higher melatonin levels but not at lower melatonin levels, there was no significant correlation between passive and cotton swab saliva collection methods.

Swabs made of materials other than cotton may not affect melatonin assay results in the same way that cotton swab collection does [15, 18]. Groschl and Rauh [18] investigated salivary cortisol levels from samples collected using certain devices, and although salivary cortisol levels obtained using the cotton swab collection method were lower than those obtained using passive collection methods, there were no significant differences in the salivary cortisol levels between passive and polyester swab collection. Weber et al. [15] have reported that polyester swabs did not affect salivary melatonin assay results. However, these earlier studies compared only mean melatonin levels between passive and polyester swab collection methods and did not evaluate relative or proportional bias. Therefore, this study examined the effects of sample collection using swabs made of non-cotton material on the salivary melatonin assay results using BA plots.

## Methods

### Participants

Eleven healthy males (age, 20–24 years) were included in the study, with their written consent. This study was approved by the ethical committee of the Faculty of Design at Kyushu University. The participants did not present with any medical condition that would interfere with the results. All the participants were nonsmokers and instructed to abstain from alcohol for 1 day and from caffeine, food, and brushing their teeth for 2 h prior to sample collection.

### Saliva sample collection

Four saliva samples were obtained at night (22:00 to 01:00 h) from each participant (44 samples in total). Because bright light acutely suppresses melatonin secretion, the saliva samples were collected under dim light conditions (< 30 lx). The samples were directly collected into clear sterile plastic tubes using sterile plastic straws. A 1-ml aliquot of each saliva sample was pipetted onto Salivette® propylene (PP)–polyethylene (PE) polymer swabs (Art. No. 51.1534.901J, Sarstedt K. K., Tokyo, Japan) (non-cotton saliva collection), and another aliquot was pipetted into clear sterile plastic tubes (passive saliva collection). All the saliva samples were centrifuged at 1500×*g* for 5 min at room temperature and then frozen at − 30 °C until further experiments.

### Salivary melatonin assay

Commercially available radioimmunoassay kits (Direct Saliva Melatonin RIA; Bühlmann Laboratories, Allschwil, Switzerland) were used to analyze the melatonin levels in duplicate, and the mean values were used for further analyses. The kit detection limit is 0.2 pg/ml, and the limit of quantification is 0.9 pg/ml. The mean intra- and inter-assay coefficients of variance were 7.9 and 9.8%, respectively.

### Statistics

A two-tailed paired *t* test was used to compare the mean salivary melatonin levels. Pearson’s correlation coefficients were calculated between the passive saliva collection and synthetic, PP–PE polymer, swab-collected samples. BA plots [17] were used to determine result agreement and bias. SPSS version 22.0 (SPSS, Chicago, IL, USA) was used to perform the statistical analyses. A *p* value < 0.05 was considered statistically significant.

## Results

Because their melatonin levels were below the quantification limit of the RIA kit (< 0.9 pg/ml), four samples were excluded from the analysis. Consequently, 40 saliva samples were analyzed.

**Fig. 1:**
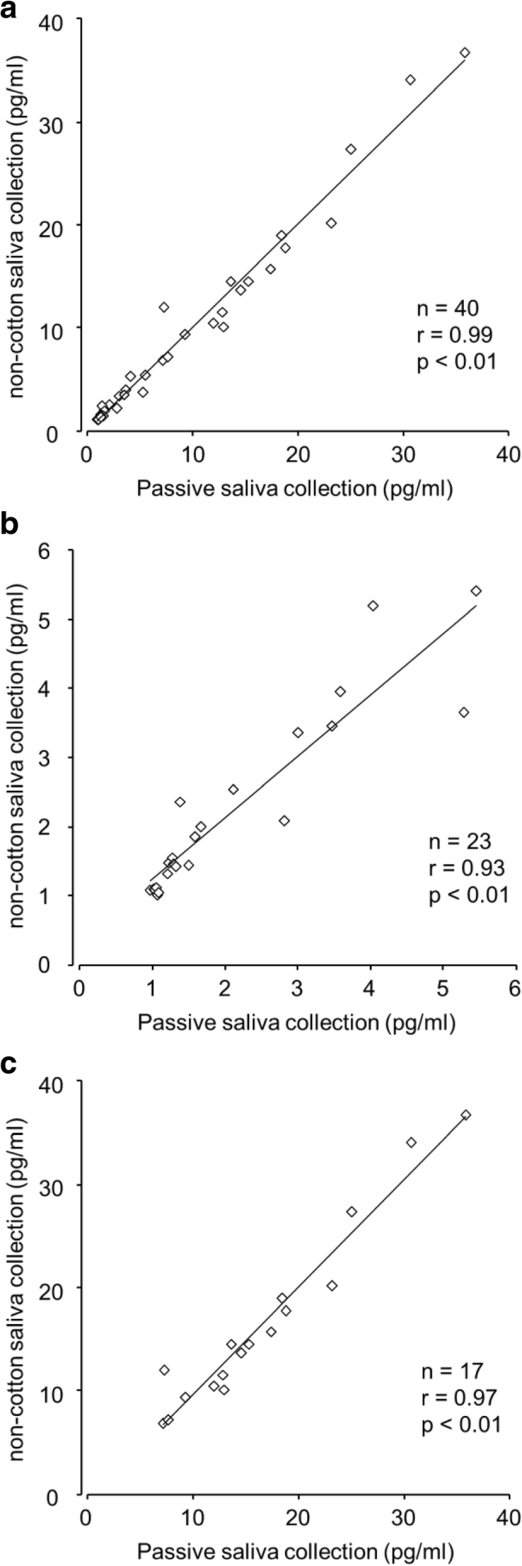
Scatter plots of melatonin levels between passive and non-cotton (PP–PE polymer) swab saliva collection for all (**a**), lower melatonin levels (**b**), and higher melatonin level (**c**) samples

Table 1 presents the mean values and standard deviations of melatonin levels, Pearson’s correlation coefficients (*r*), and lower (< 6 pg/ml) and higher (> 6 pg/ml) melatonin levels for all the samples. The mean melatonin levels at lower and higher melatonin levels did not significantly differ between passive and PP–PE polymer swab saliva collection methods. All the correlations between the collection methods were significant (Fig. 1).

**Table 1:** Pearson’s correction coefficient (*r*) and melatonin levels of passive (P) and non-cotton (PP–PE polymer) swab saliva (N) collection methods for all, lower melatonin level (< 6 pg/ml), and higher melatonin level (> 6 pg/ml) samples

	Mean and standard deviation (SD)		*r*
	P (pg/ml)	N (pg/ml)	P vs. N
< 6 pg/ml	2.11 (1.38)	2.21 (1.33)	0.93^*^
> 6 pg/ml	16.59 (8.18)	16.48 (8.75)	0.97^*^
All	8.26 (9.01)	8.28 (9.14)	0.99^*^

^*^p < 0.05

**Fig. 2:**
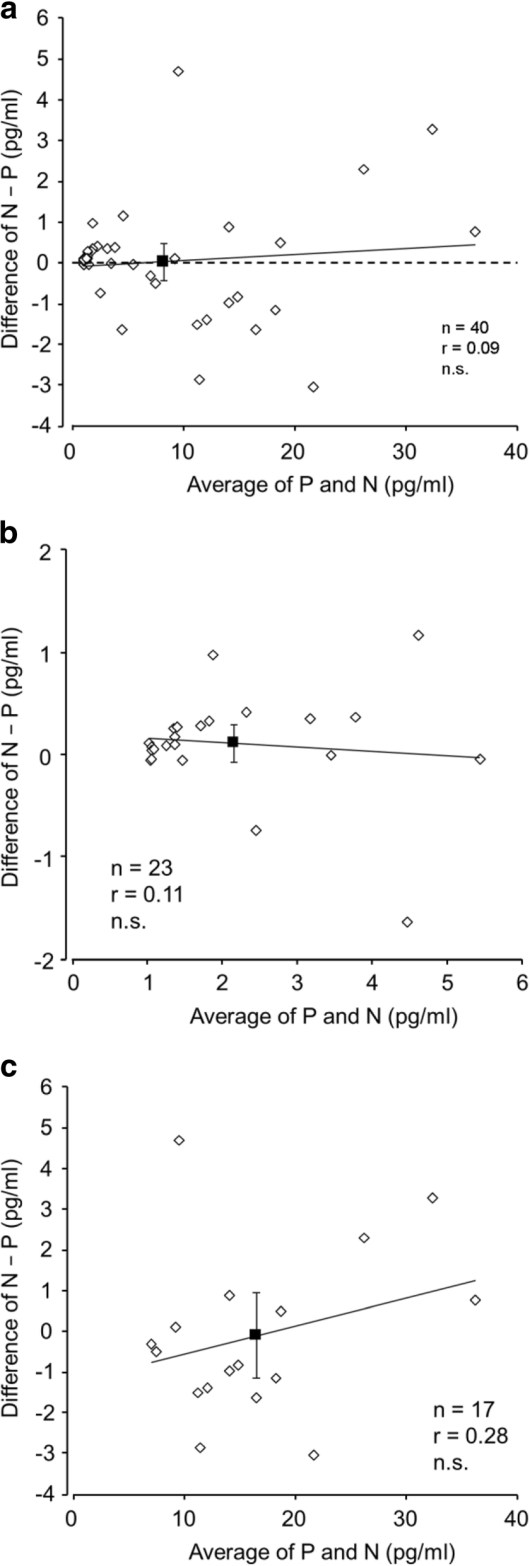
BA plots of passive and non-cotton (PP–PE polymer) swab saliva collection for all (**a**), lower melatonin level (**b**), and higher melatonin level (**c**) samples

The 95% confidence intervals (CIs) for differences in all samples at higher and lower melatonin levels between PP–PE polymer swab and passive collection methods (N − P) included zero in the BA plots (Fig. 2). The CIs for all samples ranged from 0.45 to − 0.43, ranging from 0.30 to − 0.09 at lower melatonin levels, and from 0.95 to − 1.16 at higher melatonin levels. As shown by the BA plots, averages and differences between non-cotton and passive collection methods (N − P) were not significantly correlated at both lower and higher melatonin levels. Therefore, the BA plots did not reveal any relative or proportional biases at each melatonin level.

## Discussion

No significant differences were observed in the mean melatonin levels between the passively collected and the PP–PE polymer swab-collected samples, consistent with previous results [15]. Additionally, these findings indicate a significant correlation between the PP–PE polymer swab-collected and passively collected samples at both lower and higher melatonin levels. No relative or proportional biases were detected in the BA plots. Taken together, our results demonstrate that non-cotton (PP–PE polymer) swabs do not affect salivary melatonin assay results.

PP–PE polymer swabs may be preferable to the passive drool method [19] because of a more standardized saliva flow and improved sample purity. In fact, the usage of collection devices is recommended in the operating manuals of commercially available assay kits. Furthermore, by using devices with synthetic swabs, researchers can collect saliva samples from the participants in field studies easily.

Previous studies [15, 16] have reported that the use of cotton swabs affects the salivary melatonin assay results due to interference by substances in cotton. These substances may nonspecifically link or cross-link with the specific antibody used for the assay, altering the recorded melatonin levels, particularly when melatonin levels in samples are low; however, this needs to be validated. Our results indicate that synthetic materials, such as PP–PE polymer, do not interact with the specific antibody used for salivary melatonin and cortisol assays.

Our findings indicate that PP–PE polymer swabs are useful for the assessment of DLMO and the dose response of the circadian system to light, because of the purity of the samples. However, there are some limitations to this study, because samples were not obtained directly from the mouths of the study subjects; thus, this study was considered an in vitro experiment. In contrast, Weber et al. [15] collected saliva samples using cotton swabs directly placed into the mouth of the study participants (in vivo experiment) and examined their effects on the results of the salivary melatonin assay. They suggested that the difference between the in vitro and in vivo experimental results might be due to the presence of high molecular weight proteins, such as mucins from the mouth, in the collected samples. Moreover, the selection and quality of antibodies used in the melatonin assay may affect the results [20]. The effects of sample collection using swabs made of non-cotton materials on salivary melatonin assay results should be examined in vivo and verified using additional methods, such as enzyme immunoassay.

## Conclusions

This study examined whether swabs made of non-cotton materials affect salivary melatonin assay results using the BA plot. There were no significant differences on mean melatonin level between passive and PP–PE polymer swab collection. Salivary melatonin levels from non-cotton swab collection have a high correlation with that from passive collection. The BA plot did not show a relative or a proportional bias. These findings indicated that PP–PE polymer swabs are useful for salivary melatonin assay.

## Abbreviations

BA plot: Bland–Altman plot
CI: Confidence interval
DLMO: Dim light melatonin onset
PE: Polyethylene
PP: Polypropylene
